# Family-Based Whole-Exome Analysis of Specific Language Impairment (SLI) Identifies Rare Variants in *BUD13*, a Component of the Retention and Splicing (RES) Complex

**DOI:** 10.3390/brainsci12010047

**Published:** 2021-12-30

**Authors:** Erin M. Andres, Kathleen Kelsey Earnest, Cuncong Zhong, Mabel L. Rice, Muhammad Hashim Raza

**Affiliations:** 1 Child Language Doctoral Program, University of Kansas, Lawrence, KS 66045, USA; eandres@ku.edu (E.M.A.); mabel@ku.edu (M.L.R.); 2 Language Acquisition Studies Lab, University of Kansas, Lawrence, KS 66045, USA; kkelsey@ku.edu; 3 Department of Electrical Engineering and Computer Science, University of Kansas, Lawrence, KS 66045, USA; cczhong@ku.edu

**Keywords:** specific language impairment (SLI), language, family-based, complex inheritance, multiple hit model, oligogenic, *BUD13*, splicing, RES complex

## Abstract

Specific language impairment (SLI) is a common neurodevelopmental disorder (NDD) that displays high heritability estimates. Genetic studies have identified several loci, but the molecular basis of SLI remains unclear. With the aim to better understand the genetic architecture of SLI, we performed whole-exome sequencing (WES) in a single family (ID: 489; *n* = 11). We identified co-segregating rare variants in three new genes: *BUD13*, *APLP2*, and *NDRG2*. To determine the significance of these genes in SLI, we Sanger sequenced all coding regions of each gene in unrelated individuals with SLI (*n* = 175). We observed 13 additional rare variants in 18 unrelated individuals. Variants in *BUD13* reached genome-wide significance (*p*-value < 0.01) upon comparison with similar variants in the 1000 Genomes Project, providing gene level evidence that *BUD13* is involved in SLI. Additionally, five *BUD13* variants showed cohesive variant level evidence of likely pathogenicity. Bud13 is a component of the retention and splicing (RES) complex. Additional supportive evidence from studies of an animal model (loss-of-function mutations in *BUD13* caused a profound neural phenotype) and individuals with an NDD phenotype (carrying a CNV spanning *BUD13*), indicates *BUD13* could be a target for investigation of the neural basis of language.

## 1. Introduction

An estimated 7–10% of the population who are diagnosed with specific language impairment (SLI) struggle to acquire language, despite normal hearing and no other developmental delays [1,2,3]. Humans rely heavily on language, and evidence indicates that greater language ability is associated with not only academic success but also success in social relationships [4]. Therefore, when language does not come easily or is delayed, there are long-term effects on quality of life [5,6]. Family aggregation and twin studies consistently indicate genetic transmission and a high heritability of SLI [7,8,9,10,11]. The molecular underpinnings of SLI are unknown, in part due to phenotypic variation between individuals and complex inheritance patterns.

The genetic study of SLI has revealed numerous linkage loci and candidate genes over the past 20 years. The earliest genetic studies of families with SLI revealed linkage at chromosome 16q23 and 19q13 to a test of nonword repetition (NWR) in the SLI Consortium (SLIC) families [12,13] and linkage at chromosome 13q21 to reading and language impairments in Canadian families [14]. Both groups replicated these findings in additional families, motivating further investigation [13,15,16]. We recently reported suggestive evidence of linkage at 4q31.23–q35.2, 14q11.2–q13.3, and 15q24.3–q25.3 to SLI in three independent families [17]. The 14q region was observed in a family with eight affected members (family 489); whole-exome sequencing (WES) in this family served as the starting point for the current study [17]. Notably, the 14q region overlapped with a significant paternal parent-of-origin effect in SLIC probands and the Avon Longitudinal Study of Parents and Children (ALSPAC) cohort [18]. An overlapping homozygous region on 14q was reported in consanguineous Pakistani families with language deficits, phenotyped with a vocabulary measure [19]. Additionally, among previously reported regions with an LOD scores greater than 1.2, 13q14 was observed in family 489 [17,20]. Multiple reports of overlapping loci, consistent with our findings in family 489, warranted further investigation through next generation sequencing (NGS).

NGS technologies and publicly available genetic databases offer valuable tools to uncover the biological basis of SLI, especially in family-based studies. For example, WES in a large isolated consanguineous family with language impairment from Robinson Crusoe Island identified a rare nonsynonymous variant in *NFXL1* (p.N150K) shared by multiple affected individuals [21,22,23]. The same *NFXL1* variant had a higher frequency in islanders with language impairment compared to islanders with typical language ability [22]. Sanger sequencing of all the coding exons of *NFXL1* in the SLIC probands (*n* = 117) revealed three additional non-synonymous rare variants in four SLIC probands and their family members, indicating that *NFXL1* may be an SLI risk gene in multiple populations [22]. Functional investigation of *NFXL1* revealed a high expression in brain regions associated with language development, including the cerebellum, suggesting *NFXL1* as an important target in SLI [24]. As the list of SLI candidate genes continues to grow, NGS investigations have incorporated these candidate genes into their prioritization criteria [25,26]. An NGS investigation of 43 SLIC probands revealed rare and common variants within previously suggested and novel genes, including multiple variants co-occurring in individual SLIC probands [26]. Ultimately, the results of the NGS study could be argued to support that a combination of variants, including those on previously identified genes, is more likely to explain the transmission of language impairment than any single gene, even in an individual family [26]. Together, these results demonstrated the utility of family studies and NGS in identifying protein-coding variants of a large effect in SLI.

The unknown modes of inheritance and likely variable disease penetrance poses difficulties in studying the genetics of SLI under the Mendelian modes of inheritance. The complexity of language and the SLI phenotype suggests multiple genes and gene pathways could control the expression of this poorly understood disorder [25,26,27,28]. Language acquisition is a dynamic process that occurs rapidly in typically developing children across dimensions (grammar, vocabulary, and discourse) in both the receptive and expressive domain [29]. Individuals with SLI are known to show specific deficits in receptive and expressive grammar and vocabulary, although there is variance within individuals for these dimensions [29,30,31]. Therefore, while the lack of replication of the reported SLI candidate genes has been attributed to inconsistency in phenotype measurement, it could also be the case that the genes suggested previously explain a portion of the dynamic process of language acquisition or the complexity of skills that represent language ability. A combination of genes with variable effect size may explain the complexity of language [26,32].

**Figure 1:**
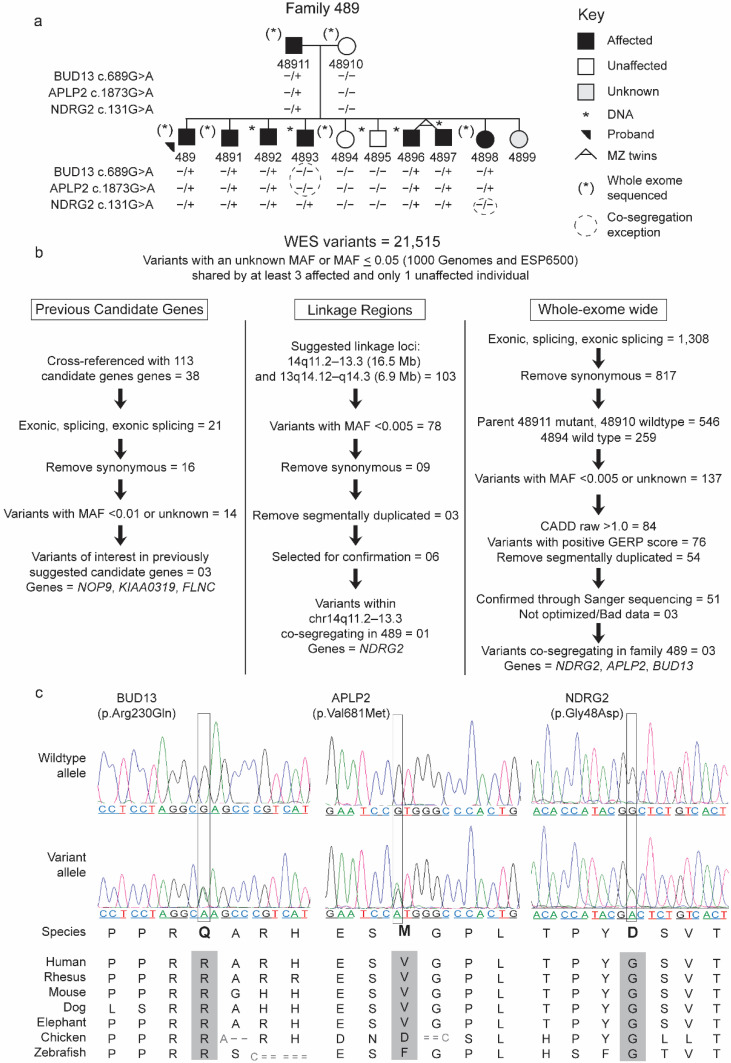
Prioritization of exome variants in family 489. (**a**) The family 489 pedigree and co-segregation of the three identified variants. (**b**) WES variant filtering workflow. WES—whole exome sequencing; MAF—minor allele frequency; CADD—combined annotation-dependent depletion; GERP—genomic evolutionary rate profiling. (**c**) Electropherograms showing the variant and wildtype alleles for the three identified variants with their conservation across vertebrates.

The current study was motivated by our ascertainment of a family with many cases of SLI and was designed to identify variants of interest from the WES that may give further insight into the genetic pathways involved in SLI. We used WES data from a single family (ID: 489), with a follow-up analysis of prioritized variants in additional probands with SLI. All of the individuals were collected as part of a larger cohort of families with SLI at the University of Kansas (KU Cohort; Figure 1a). The KU cohort has been followed longitudinally since 1993, and the criteria for SLI have remained constant over the study period [30] (see criteria in Materials and Methods Section 2.1.3). We first narrowed the WES output from family 489, by reducing the list of variants from thousands to those most likely to be associated with the phenotype, through filtering. Our filtering workflow utilized available bioinformatic tools and genomic databases based on guidelines for identifying true disease-causing variants from the sequencing data [33]. We prioritized rare non-synonymous exome variants in candidate genes previously suggested for language and related phenotypes in the linkage regions previously reported in family 489 (14q and 13q) [17], and variants in novel genes not previously suggested. Finally, to estimate the significance of rare variants at the gene level, we performed Sanger sequencing of all coding exons of three identified novel candidate genes in the larger KU cohort.

## 2. Materials and Methods

### 2.1. Participants

#### 2.1.1. Family 489

Family 489 is a 12-person monolingual English-speaking Caucasian family, part of a larger ongoing longitudinal study in the Language Acquisition Studies (LAS) Lab directed by Dr. Mabel L. Rice at KU. This family was recruited from a school speech pathology caseload, under a study approved by the KU institutional review board (IRB #8223), with appropriate informed consent obtained from all of the participants. Behavioral data and DNA were obtained from both parents and nine children. Sibling 4899 was too young at the time of final data collection to provide DNA or complete behavioral assessment (Figure 1a).

#### 2.1.2. Additional Participants

**Figure 2:**
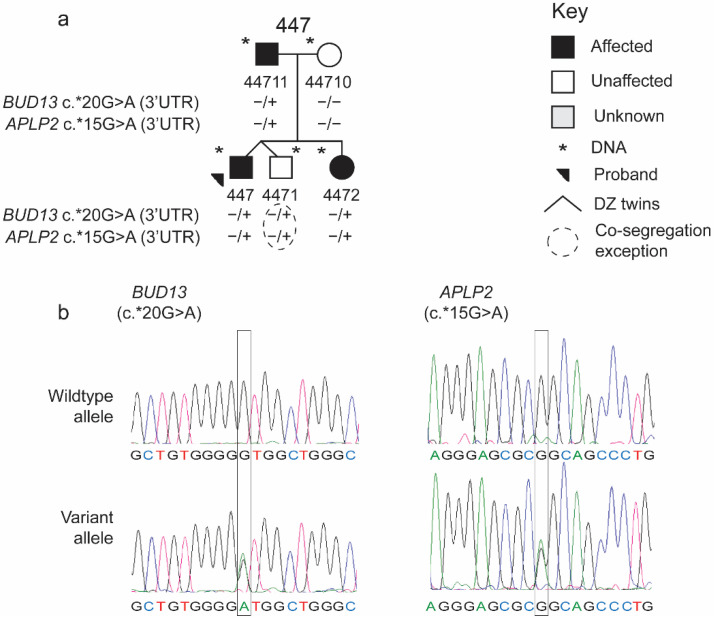
*APLP2* and *BUD13* 3′UTR variants co-segregating in family 447. (**a**) The family 447 pedigree and co-segregation of the two identified variants. (**b**) Electropherograms showing the variant and wildtype alleles for the two identified variants.

We selected unrelated individuals with SLI from the proband-ascertained families (*n* = 175) to estimate the significance of rare variants in the identified novel candidate genes and previously reported candidate genes. The unrelated individuals include 170 probands, who entered the study based on the proband entrance criteria described below, a half cousin of one proband, and four married-in individuals. The distant relation of a half cousin and the multiple affected individuals in the extended family warranted the inclusion of two individuals from this family. The self-reported race and ethnicity percentages for the additional participants (*n* = 175) were as follows: white, 78.3%; multiracial, 13.2%; American Indian, 3.4%; Black, 1.7%; and not reported, 3.4%. Hispanic ethnicity was reported by 9.7% of the sample, 89.2% not Hispanic, and 1.1% not reported. Additionally, we selected available family members (*n* = 74) of the 23 unrelated individuals with SLI for Sanger sequencing, in which rare variants were identified in the novel candidate genes or the same variant(s) was identified in the previously reported candidate genes (Figure 2 and Figures S1–S3).

#### 2.1.3. Phenotype

SLI in the current study was defined by inclusionary and exclusionary criteria, including average nonverbal intelligence (NV-IQ), as defined by a standard score > 85 on the Columbia Mental Maturity Scale for children between the age of 3.6 to 6.11, or an age-appropriate standardized Wechsler Intelligence Scale for individuals aged 7.0 and older, but with a standard score ≤ 85 on an age-appropriate standardized omnibus language measure [17,30,34,35]. In accordance with the larger ongoing longitudinal study, community ascertained probands entered the study as children with SLI by meeting the following four entrance screening criteria: (i) no cognitive impairment (NV-IQ > 85); (ii) no hearing loss; (iii) no other diagnoses of developmental delay, neurological disorder, or autism at the initial time of assessment (based on parent report); and (iv) intelligible speech. There were no probands or family members with Childhood Apraxia of Speech (CAS). In addition, the probands and family members were monolingual native speakers of English and were screened for nonstandard dialects. A full list of the phenotype measures (including language, reading, and intelligence) collected as part of the ongoing longitudinal study is described in an earlier publication by Rice and colleagues [32].

In the current study, affection status was assigned categorically and was determined based on the performance on an age-appropriate standardized omnibus language measure. Individuals with a standard score ≤ 85 were categorized as affected. Unrelated individuals with SLI (*n* = 175) from the proband-ascertained families scored ≤ 85 on an age-appropriate standardized language measure at the first time of assessment. The affection status of the 74 additional family members from the 23 proband-ascertained families was determined following precedents in previous longitudinal studies for assigning affectedness status. Family members were classified as affected based on their lowest standardized omnibus language score across all occasions of measurement. Individuals who never scored in the affected range across times of measurement (i.e., standard scores were consistently > 85) were classified as unaffected. For details concerning the administration and the editions of the omnibus measures used, see an earlier publication by Andres and colleagues [17].

### 2.2. Genetic Analyses

#### 2.2.1. DNA Collection and Preparation

Saliva samples/buccal swabs were collected using the Oragene-Discover OGR-500 or OGR-575 Kits (DNA Genotek, Oragene, Ottawa, ON, Canada). DNA was purified from the samples according to the manufacturer’s instructions.

#### 2.2.2. Whole-Exome Sequencing and Data Analysis

In this study, we analyzed the WES data from four affected and two unaffected members of family 489 (both parents, the proband, and three siblings; Figure 1a,b). Exome capture was performed using the Nextera Rapid Capture Enrichment kit (expanded; includes untranslated genomic regions; San Diego, CA, USA) from Illumina (Illumina Nextera DNA exome; San Diego, CA, USA), which covers ≥ 98% of RefSeq, CCD, and Ensemble coding content of genes. The captured exomes were sequenced using the Illumina HighSeq instrument with paired-end sequencing at the University of Nebraska Medical Center Genomics Core Facility. The high-quality sequencing data were mapped to the human reference genome (hg19) using the Burrows—Wheeler Aligner (BWA) and Genome Analysis Toolkit (GATK) best practices pipeline [36,37]. The resulting VCF file was processed to identify coding and non-coding variants after removing low quality calls. Low-quality calls did not meet at least one of the following criteria: QUAL ≥ 50 (quality score), VQSLOD ≥ 0 (variant quality score log-odds of being a true variant versus being false based on the trained Gaussian mixture model), DP ≥ 5 (read depth) for all individuals in a family, and QD > 5 (quality by depth = QUAL score divided by allele depth).

#### 2.2.3. Prioritization of Rare Variants in the WES

We established a priori criteria to prioritize rare protein coding and splicing variants in the WES output. Rare variants were defined as those with a minor allele frequency (MAF) ≤ 0.005 in the 1000 Genomes Project and NHLBI GO Exome Sequencing Project 6500 (ESP6500) databases, or an unknown MAF. At the time of WES annotation (prior to 2016), only 1000 Genomes Project and ESP6500 were available for annotation. However, the frequencies were acquired from the Genome Aggregation Database (gnomAD) version 2.1.1 exomes for the variants of interest (Table 1, Table 2, Table 3, Table 4 and Table 5) [38,39,40]. We prioritized variants under three workflows: (1) previous candidate genes, (2) linkage regions, and (3) whole-exome wide (Figure 1b). The three filtering workflows were applied to 21,515 variants (intronic, exonic, splice site, UTRs, intergenic, and indels) that were present in ≥ three affected individuals and ≤ one unaffected individual and had a MAF less than 5% in the 1000 Genomes Project or ESP6500 (Figure 1b).

We established a list of candidate genes previously reported for language and related phenotypes (113 genes; Table S8), in order to initially evaluate WES variants on previously reported candidate genes prior to prioritizing variants on novel genes, as recommended by MacArthur et al. [33]. Our list combined candidate genes from investigations by Chen and colleagues (2017) and Mountford and colleagues (2019), which similarly established lists prior to evaluating NGS data, as well as genes from the most recently published comprehensive candidate gene review by Guerra and colleagues (Table S8) [25,26,33,41,42]. Guerra and colleagues’ review comprehensively reports 83 genes previously suggested for multiple disorders, including SLI or developmental language disorder (DLD), speech sound disorder (SSD), childhood apraxia of speech (CAS), stuttering (ST), aphasia (AP), dyslexia (DL), and autism spectrum disorder (ASD). Our rationale for including all 83 genes in the Guerra review is three part, namely: these phenotypes can often be comorbid, especially reading disorder/dyslexia with SLI [32,43,44,45]; across studies, these phenotypes have been evaluated with varying measurements, which could mean variability in language has been captured in one of these previous reports; and the other phenotypes could share common gene pathways with SLI, meaning family 489 could also carry variants on these genes. Note that both Chen and colleagues and Mountford and colleagues also included genes on their shorter lists (19 and 34 genes, respectively) that had been associated with language related phenotypes, like ASD. Ultimately, including all of these genes in our list ensured we did not overlook any previous reports. Additionally, we included the novel genes reported by Chen and colleagues, in which SLIC probands carried more than one variant or carried a stopgain variant, which accounted for 15 of the genes on our list [26]. We added one gene to our compiled candidate gene list, *ZNF277*, identified in a girl with SLI [42].

Next, we prioritized variants within the suggestive linkage regions previously reported in family 489 on chromosomes 13q and 14q (Figure 1b) [17]. The observed linkage in this family did not reach genome wide significance, indicating other regions may also contain causal variants. Therefore, we also prioritized any novel rare nonsynonymous variants whole-exome wide.

Rare variants were prioritized whole-exome wide based on the following criteria: (i) classified as exonic, splicing, exonic splicing, or insertion/deletions (indels); (ii) carried by the affected father, but not unaffected mother; (iii) an unknown raw Combined Annotation Dependent Depletion (CADD) score or score > 1; (iv) a positive genomic evolutionary rate profiling (GERP) score; and (v) located within a non-segmentally duplicated genomic region (Figure 1b). CADD scores indicate the predicted deleteriousness of single nucleotide variants (SNV) or indel variants in the human genome [46]. GERP scores indicate the conservation of nucleotides among multiple species and can range from −12.3 to 6.17, wherein a higher score indicates the nucleotides are more conserved [47].

#### 2.2.4. Identification of Candidate Genes, Confirmation, and Significance Testing

The filtered rare variants were confirmed via Sanger sequencing in the six individuals used for WES. Then, confirmed rare variants were Sanger sequenced in the five additional available family members and were evaluated for co-segregation with SLI in family 489. Due to the unknown penetrance of SLI, perfect co-segregation was not expected. In family 489, variants were considered to follow co-segregation when confirmed in at least seven affected individuals but zero unaffected individuals, or confirmed in all eight affected members but also present in one unaffected child. Although there were three unaffected members of family 489, one of them was the mother of the proband; rare variants observed in the unaffected mother were not used for confirmation in the unrelated individuals with SLI (unless in a previously suggested candidate gene). Genes with rare variants meeting the a priori co-segregation criteria in family 489 were selected as candidate genes. All coding exons of the identified novel candidate genes and the variant locations of each of the variants identified in the previously reported candidate genes were Sanger sequenced in unrelated individuals with SLI (*n* = 175). We Sanger sequenced the variants in the available family members of unrelated individuals in which rare variants were observed (*n* = 74).

MacArthur and colleagues have provided a classification of evidence that can be assessed to implicate either a gene or a variant in the transmission of a disorder [33]. At the gene level, gene burden calculation can provide evidence implicating a gene in the transmission of a disorder. In the current study, we estimated the significance of the rare variants at the gene level using the Significance of Rare VAriants (SORVA) program. Gene-based queries were run using the rate of LOF (loss of function) and missense variants in the global population and the European population from the 1000 Genomes Project. The MAF was set to 0.005 and the total number of the sequenced genes was set to three [48]. The total number of unrelated individuals with SLI was reduced by one (*n* = 174) for the SORVA analysis because two individuals (distantly related half cousins) were originally selected for the follow-up Sanger sequencing from one proband-ascertained family.

Genetic evidence at the variant level included MAFs from gnomAD v2.1.1 exomes, co-segregation of the variant in the probands’ family (our a priori criteria of one exception allowed was maintained for these families), and the observance of the variant in multiple probands [33]. Additionally, variant level informatic evidence included conservation scores, in silico prediction scores, and predictions about protein structure changes due to the variant. Conservation was measured by GERP scores acquired from the UCSC human genome browser (hg19) [47]. We reported five in silico prediction scores for the variants identified via Sanger sequencing, and each was acquired by inputting the location of the change into online tools. PROVEAN (Protein Variation Effect Analyzer) and SIFT (Sorting Intolerant from Tolerant) scores were both acquired from the J. Craig Venter Institute (JCVI) PROVEAN website (http://provean.jcvi.org/index.php; accessed on 26 October 2021) [49,50]. PolyPhen-2 (Polymorphism Phenotyping v2) scores, Mutation Assessor scores, and MutationTaster2 scores were acquired from their respective websites (http://genetics.bwh.harvard.edu/pph2/bgi.shtml (accessed on 26 October 2021) [51,52,53]; http://mutationassessor.org/r3/ (accessed on 26 October 2021) [54]; and https://www.mutationtaster.org/ (accessed on 26 October 2021) [55]. MutationTaster2 scores were based on hg19 [55]. These scores were acquired on or before 26 October 2021. Finally, we used the HOPE (Have yOur Protein Explained) server, which provides information about the structural changes that will occur in response to the variant allele changing a protein (website: https://www3.cmbi.umcn.nl/hope/; accessed on 26 October 2021) [56].

We used genetic and informatic evidence at the variant level recommended by MacArthur et al. to classify the predicted causality of each reported variant as pathogenic or benign (as defined in the Table 5 note). However, these predictions still need to be tested.

**Table 5:** Summary of evidence supporting causality of variants identified in family 489 and probands with SLI.

		Fam 489 Variants						Additional Variants												
		Previous Candidates			Co-Segregating			*NDRG2*	*APLP2*			*BUD13*								
**Evidence** **Class**	**Evidence**	***KIAA0319*** rs113411083	***FLNC*** rs202223616	***NOP9*** rs183868211	***NDRG2*** rs11552412	***APLP2*** rs370970986	***BUD13*** rs139478949	rs779725845	rs1063201	chr11:129992279	rs201861910	rs35585096	rs116087150	rs1467808735	rs144776650	rs11216131	rs1427011653	rs145410701	rs61730763	rs145906707
Genetic	MAF ≤ 0.05	+	+	−	+	+	+	+	+	+	+	+	+	+	+	+	+	+	+	+
	Co-segregation	−	−	−	+	+	+	−	−	−	+	−	−	−	−	−	−	−	−	+
	≥1 proband	−	−	+	−	−	−	−	−	−	−	+	−	−	+	−	−	−	−	+
Informatic	Positive GERP Score	+	+	+	+	+	+	+	−	+	−	+	+	+	−	+	−	−	+	+
	Total # of damaging in silico scores	2	1	0	4	2	4	2	0	2	NA	2	4	4	1	5	0	0	3	NA
	HOPE output/AA change																			
	Size	∧	∧	∨	∧	∧	∨	∧	∧	∧	NA	∧	∨	∨	∨	∨	∧	∨	∧	NA
	>Hydrophobic	+	−	+	+	−	−	+	−	−	NA	+	+	+	−	+	−	+	−	NA
	Charge change	pos	neg	pos	neu	NC	pos	NC	NC	neg	NA	NC	pos	neu	pos	pos	NC	NC	NC	NA
		to	to	to	to		to			to			to	to	to	to				
		neu	pos	neu	neg		neu			pos			neu	neg	neu	neu				
	Causality	P	B	B	P	P	P	P	B	P	NA	P	P	P	B	P	B	B	P	NA

MAF—minor allele frequency; ‘+’ = yes, ‘−’ = no; NA—not applicable; ∧ bigger, ∨ smaller; pos— positive; neu—neutral; NC = no change; P—pathogenic; B —benign. HOPE (Have yOur Protein Explained) output (mutant analysis server explaining structural changes due to protein change)—size change: a bigger mutant amino acid leads to bumps in structure, a smaller mutant amino acid leads to loss of interactions with other amino acids. More hydrophobic: a more hydrophobic mutant amino acid leads to a loss of hydrogen bonds and disruption in the folding of the amino acid. Charge change: if the mutant amino acid becomes neutral it leads to a loss of interactions with other amino acids; if a charge is introduced (from neutral to a charge) or the opposite charge is introduced (from one charge to the other) the mutant amino acid will repulse other amino acids [56]. Causality classifications: pathogenic (P) = (1) MAF < 0.05 (in gnomAD v2.1.1 exomes) AND (2) positive GERP score (conserved) AND (3) > 2 damaging in silico prediction scores AND (4) EITHER co-segregating OR carried by > 1 proband OR some significant change to amino acid structure; benign (B) = (1) MAF > 0.05 OR (2) negative GERP score (conserved) OR (3) < 2 damaging in silico prediction scores.

## 3. Results

In total, four affected and two unaffected individuals were used for the analysis of WES in one family (see Methods). Under the candidate gene filtering workflow, we observed non-synonymous variants in three previously reported candidate genes (Table 1), while the suggestive linkage regions filtering workflow prioritized one variant that was also prioritized under the whole-exome wide filtering, along with two additional variants (Table 2). In total, six variants and the coding regions of the three novel identified candidate genes were prioritized for Sanger sequencing in the additional probands with SLI (*n* = 175).

Within the list of candidate genes previously suggested for language and related phenotypes, we observed variants of interest on *KIAA0319*, *FLNC*, and *NOP9* (Table 1). Sanger sequencing confirmed these three variants in family 489. However, none of the variants were co-segregated in family 489 (Table 1). The variants in *KIAA0319* and *FLNC* were not observed in the 175 unrelated individuals with SLI (Table 1). The variant in *NOP9* is within the 14q linkage region mapped in family 489 [17]; according to the exome sequencing, this variant was inherited from the unaffected mother and the affected father did not carry this variant. The *NOP9* variant was only prioritized under the candidate gene filtering workflow (Figure 1b), because the MAF of this variant exceeded the threshold for the whole-exome wide criteria (1000 Genomes = 0.0051 and ESP6500 = 0.0068). Note, the MAF in gnomAD for the global population (0.00936) also falls under the MAF threshold (0.01) for the previous candidate gene filtering workflow (Figure 1b and Table 1). According to the segregation analysis in family 489, the Sanger sequencing confirmed that the *NOP9* variant was inherited from the unaffected mother (Figure S3). The follow-up sequencing of the 175 probands with SLI showed an additional five probands carried the same variant, c.62G > C: p.Arg21Pro, in *NOP9* (Figure S3). Although the amino acid is conserved among vertebrates, the effect of the *NOP9* variant was predicted as benign by all five in silico predictions and gnomAD, and the ALSPAC cohort showed a higher MAF (0.01–0.02) in Europeans (Table 1 and Table 5). Additional variants met the candidate gene filtering workflow criteria. Ten of these variants were on *MUC6* (Figure 1b and Table S7); MUC family genes are commonly observed in NGS data, regardless of the phenotype under investigation; this was also noted by Chen and colleagues, so we did not confirm the *MUC6* variants via Sanger sequencing [26]. The remaining variant was in *NCOR1*, and was not sequenced because we found the variant was a common polymorphism according to the MAF in gnomAD v2.1.1 exomes (global MAF = 0.1645) despite the unknown MAFs, according to the annotation of WES data with the 1000 Genomes Project and ESP6500 [38].

Next, we prioritized 103 variants within the suggestive linkage regions at chromosomes 13q and 14q. Rare non-coding variants (intronic, UTRs, ncRNAs, and intergenic), synonymous variants, and rare variants in segmentally duplicated regions were filtered out, leaving six rare protein coding variants in the linkage regions. The prioritized six variants were confirmed through Sanger sequencing and their co-segregation was evaluated in family 489. We observed one variant c.143G > A: p.Gly48Asp, in *NDRG2* (NM_201535) co-segregating in family 489 (one affected individual, 4898, did not carry the mutant allele; Figure 1a). We prioritized *NDRG2* within the linkage regions as a candidate gene for follow-up genetic analysis in additional unrelated individuals with SLI, while the other five variants did not show co-segregation in family 489 and were not considered for follow-up (Figure 1b).

Finally, rare variants were filtered whole-exome wide in the WES data. First, non-coding (intronic, UTRs, ncRNAs, and intergenic) and synonymous variants were removed, leaving 817 non-synonymous, stopgain, splicing, and indel variants. Then, under the dominant model, assuming that the mutant allele was inherited from the affected parent (ID: 48911; Figure 1a,b), 546 variants remained. Then, we removed the variants identified in the unaffected sibling (ID: 4894; Figure 1a,b), resulting in 259 remaining variants. Next, we filtered out common polymorphic variants (MAF ≥ 0.005), leaving 137 variants for further analyses (Table S1). Finally, rare variants observed in segmentally duplicated regions, variants with CADD scores less than 1.0, and variants with negative GERP scores were eliminated (Figure 1b). Altogether, these criteria prioritized 54 rare variants shared by at least three affected individuals (sometimes all four). We confirmed 51 variants through Sanger sequencing in all members of family 489. Primer pairs for three variants could not be optimized. Three of the remaining 51 confirmed rare variants showed the a priori co-segregation criteria in family 489: c.689G > A: p.Arg230Gln in *BUD13* (NM_032725.4), c.2041G > A: p.Val681Met in *APLP2* (NM_001642), and c.143G > A: p.Gly48Asp in *NDRG2* (NM_201535; Table 2 and Figure 1). The variant in *NDRG2* was also identified in the suggestive linkage region, as described above. The amino acids at the sites of the three variants are strongly conserved in vertebrates (Figure 1b). These variants were not observed in the 175 unrelated individuals with SLI (Table 2). In summary, analyses of the WES data in family 489 provided evidence for three new candidate genes (*NDRG2*, *APLP2*, and *BUD13*) that were not previously implicated in SLI. We then Sanger sequenced all coding exons of *NDRG2*, *APLP2*, and *BUD13* in 175 unrelated individuals with SLI.

Sequencing all coding exons of *NDRG2* in unrelated individuals with SLI (*n* = 175) revealed one additional non-synonymous rare variant (rs779725845, c.59C > T (p.Thr20Met)) in one individual with SLI. The variant’s MAF is 0.00002 in the 1000 Genomes Project and gnomAD databases. This variant was found to be conserved among most vertebrates and was predicted to be damaging according to SIFT and PolyPhen-2 (Table 5). Only the DNA of the proband’s mother was available and Sanger sequencing did not reveal this variant in the mother (data not shown). According to our classification criteria for pathogenicity, the variant is predicted to be pathogenic (Table 5).

The sequencing of all coding exons of *APLP2* in unrelated individuals with SLI (*n* = 175) identified two additional non-synonymous variants (c.660T > G (p.Asp220Glu) and c.793G > A (p.Glu265Lys) and one variant in the 3′UTR (c.*15G > A) in three individuals (individual IDs: 447, 463, and 434; Table 3). These variants were sequenced in 15 additional family members of these three individuals (Figure 2 and Figure S2). Two variants (c.*15G > A and c.793G > A) were observed in the majority of affected family members, and unaffected parents did not carry the variant (Figure 2 and Figure S2). One variant (c.660T > G) was not found in any other family members of the proband, although the DNA sample of the proband’s father was not available (Figure S2). The identified *APLP2* variants were rare in the 1000 Genomes Project and gnomAD databases. According to SIFT and Mutation Taster, one non-synonymous variant is predicted to be damaging or disease causing and the other variant is predicted to be benign or neutral (Table 3). According to our classification criteria for pathogenicity, one variant (rs1063201) is predicted to be benign, while the novel variant (chr11:129992279) is predicted to be pathogenic, and no claim was made about the 3′UTR variant (Table 5).

Sequencing of *BUD13* in unrelated individuals with SLI (*n* = 175) revealed nine other rare variants in 15 unrelated individuals with SLI (Figure S1). These nine rare variants included non-synonymous variants and a variant in the 3′UTR (Table 4 and Figure S1), but no rare indels, frameshifts, splicing, or stop codon variants were observed. Three of the nine variants were observed in more than one individual with SLI; c.45G > T was observed in four individuals, c.581G > A was observed in three individuals, and *20G > A in the 3′UTR was observed in two individuals (Table 4 and Figure S1). The nine *BUD13* variants were sequenced in 49 additional family members of these 15 individuals. Three rare variants (described above) were aggregated in multiple families with SLI. According to our classification criteria, five of the nine *BUD13* variants were predicted to be pathogenic, while three were predicted to be benign, and no prediction was made about the 3′UTR variant (Table 5).

Genetic evidence (gene burden) at the gene level was measured for each Sanger sequenced gene using SORVA [48]. The rate of identified rare variants in *NDRG2* and *APLP2* in our SLI sample did not reach genome-wide significance. A gene-based query of rare missense or loss of function variants in *BUD13* indicated that variants were present in 3.83% of individuals in the global population (Bonferroni corrected *p*-value < 0.01) and in 5.57% of the European population (Bonferroni corrected *p*-value > 0.05). The rate of identified *BUD13* rare variants in individuals with SLI reached genome-wide significance when compared to the global population (1000 Genomes Project estimate).

## 4. Discussion

We used a family-based approach in conjunction with WES and identified rare variants of plausible large effect shared among individuals with SLI in multiple proband-ascertained families. Rare non-synonymous, splice site, and indel variants were prioritized, including targeted prioritization in the linkage regions mapped to family 489 [17], and in the pre-determined list of previously suggested candidate genes in language and related phenotypes (Table S8). Our a priori variant prioritization criteria in the WES data identified and confirmed co-segregation of rare variants in three genes with likely pathogenic roles—*BUD13*, *APLP2*, and *NDRG2* in family 489. Note that the *NDRG2* variant was located within the suggestive 14q linkage region in family 489. Linkage was not detected on chromosome 11, although the *BUD13* and *APLP2* variants (both located on chromosome 11) were co-segregated in family 489 [17]. The follow-up sequencing of all coding exons of these three genes in additional probands with SLI identified a higher rate of rare variants in *BUD13* (9) compared to the rate of such variants in *APLP2* (3) and in *NDRG2* (1). Three variants (c.64G > T, c.581G > A, and c.*20G > A) were observed in multiple unrelated individuals with SLI and were prevalent in their family members (Figure S1), suggesting variants in *BUD13* may increase the risk of SLI.

Chen and colleagues’ prediction of the complex susceptibility model was supported by their observation of WES variants in multiple genes in individual SLIC probands, but the co-segregation could not be examined due to the lack of consistent and/or precise phenotyping of the parents [26]. Parents in the SLIC cohort completed an NWR task, but did not complete a standardized language measure, while the probands’ performance on the CELF-R indicated their severe SLI status [26]. In the current study, most parents completed a standardized omnibus language measure. Previous analysis of performance on the CELF-3 in the larger KU cohort database indicated stability in the standard scores over time, even up to age of 30+ in both affected and unaffected individuals who had been assessed multiple times prior to and after the age of 18 [17]. Importantly, the language abilities were known for both parents in family 489 and 447, the families in which co-segregation was observed with rare variants in *BUD13* and *APLP2* (Figure 1 and Figure 2). The observation of multiple variants in an individual or a family suggests a possible additive effect of multiple variants to the severity and complexity of the disorder. Our data provide support to the hypothesis of the polygenic effect, which need to be tested in a larger sample.

Complex disorders, like SLI, have shown a variable behavioral expression across individuals, although trait variance can be reduced in family-based investigation [57]. In the current study, the co-segregation for each variant identified in family 489 had one exception: one affected individual did not share the mutant alleles, which could be a phenocopy. Interestingly, the affected sibling 4893 did not share the mutant alleles in *BUD13* and *APLP2*, while the mutant allele in *NDRG2* was not found in the affected sibling 4898 (Figure 1a). Similarly, additional *BUD13* and *APLP2* variants showed co-segregation in a proband-ascertained family (family 447), except for one unaffected twin that shared these variants (Figure 2). Similar patterns were observed in language and related phenotypes, and other genetic disorders like intellectual disability, which demonstrate deviation from a simple Mendelian inheritance pattern [26,27,58,59].

The context of previously suggested candidate genes and linkage regions is crucial in order to acknowledge in the continued genetic investigation of variants in any phenotype [33]. In the current study, we observed 14 exome variants in five candidate genes previously reported for language and related phenotypes in our filtered list (*KIAA0319*, *NOP9*, *FLNC*, *NCOR1*, and *MUC6*; Table S7). The majority of the filtered candidate gene variants were located on *MUC6*. Variants on the *MUC6* gene were reported previously in SLIC probands, but are more likely to be a false positive (as described by Chen and colleagues and in our Results section above) [26]. Two variants on previously reported candidate genes warrant further discussion: rs113411083 on *KIAA0319* and rs183868211 on *NOP9* (Table 1). The previous targeted linkage and SNP association study of the KU cohort (focused on loci implicated in reading impairment) showed linkage to three endophenotypes on multiple SNPs on *KIAA0319*: the omnibus phenotype (used in the current study) and grammar and reading phenotypes [32]. The combination of previous findings in the larger KU cohort and the current study’s variant level evidence of a possible causal role of rs183868211 in SLI may indicate that *KIAA0319* variants contribute to a broader risk for language and reading impairments within this population. *NOP9* is a previously suggested candidate within the 14q linkage region in family 489 [17,18,60]. We confirmed a non-synonymous *NOP9* variant (c.G62C:p.Arg21Pro; rs183868211) via Sanger sequencing in family 489 and five additional proband-ascertained families (Table 1 and Figure S3). The *NOP9* variant has a likely benign role but could be a genetic modifier in family 489. It was inherited to the affected siblings from the unaffected mother in the presence of other variants (*BUD13*, *APLP2*, and *NDRG2*). The role of genetic modifiers was observed in families with childhood-onset cardiomyopathy, autism spectrum disorder, and Bardet—Biedl syndrome [61,62,63,64,65]. This could be something to consider in future analyses of WES data in other families with SLI, especially when considering variants in previously suggested candidate genes.

We filtered out UTR variants in the WES analysis because it is challenging to test or predict the effect of these variants, but we did cover portions of the UTRs during Sanger sequencing. Notably, we observed 3′UTR variants in *BUD13* and *APLP2*, both located on chromosome 11q and co-segregating (with one exception) in a single proband family (ID: 447; Figure 2). Although co-segregation of both variants in the same family does not qualify them as causal variants for SLI, the functional consequences of these variants should be further investigated in SLI. Recent reports indicate 3′UTR regions are essential in regulating gene expression by providing the binding sites to microRNAs, and the variants in these regions were implicated specifically in SLI, as well as other neurological disorders like intellectual disability [66,67].

While multiple genes have been suggested as candidates for language and related phenotypes, most have not been replicated previously. However, some suggested candidate genes are involved in shared biological processes, such as transcription factors (*FOXP2* and *NFXL1*) [22,24,68,69], endocytic pathways and intracellular trafficking (*GNPTAB*, *NAGPA*, *GNPTG*, and *AP4E1*) [70,71,72,73], and RNA processing or splicing mechanisms (*NOP9* and *RBFOX2*) [18,60,74,75,76,77]. We identified *BUD13* as a novel candidate, which is an important component of the retention and splicing (RES) complex. *BUD13* was studied using loss of function mutations in animal models, disrupting the RES complex [78]. Increased cell death and a reduction of differentiated neurons in the zebrafish models showed the important role of an intact RES complex in early vertebrate development and neural functions [79]. Transcriptomic analysis of RES complex genes in the mutant zebrafish identified the features of RES dependent introns [79]. Although no rare variants in *BUD13* are reportedly causative in other genetic disorders, a copy number variant (CNV) investigation revealed a deletion in chromosome 11 spanning *BUD13* in an individual with an NDD phenotype [80]. The same individual showed a deletion in chromosome 9 encompassing *PTPRD*, suggesting an additive effect in this patient [80]. Additionally, the risk of developing a metabolic syndrome was associated with *BUD13* through case-control studies [81,82]. There is more to uncover about the function of this gene, but we predict that missense *BUD13* variants in individuals with SLI may cause a subtle change in the protein function affecting the alternate splicing mechanisms of other genes.

Alternate splicing produces a diversity of proteins by keeping and or removing alternate exons from a single pre-messenger RNA. Intron retention (IR) is a process controlled by the RES complex [78]. During IR, unspliced introns are retained in messenger RNAs (mRNA), which then determines the fate of mature mRNAs [83,84]. It is suggested that IR containing messenger RNAs (IR-mRNAs) may trigger multiple molecular mechanisms. For example, IR-mRNAs may encounter premature termination codons, resulting in nonsense mediated decay mechanisms (NMD) in the cytoplasm or activating micro-RNA mediated mRNA degradation [83]. These IR-mRNAs may be detained in the nucleus for cleavage or be exported to the cytoplasm for the translation of novel functional isoforms [83]. IR events are widely distributed in human and mouse brains. The enrichment of intron retaining specific mRNAs in Alzheimer’s disease (AD) revealed a functional association of IR with AD [85]. Although there is not much known about the precise mechanisms of the RES complex in regulating gene expression, the zebrafish model suggests it plays a role in brain development and neural survival [79]. Regulation of mRNAs through splicing and other mechanisms like non-coding RNAs are proposed in LI and other NDDs [66,86,87]. The CNVs spanning *BUD13* and *PTPRD* identified in a patient with NDD suggest a multiple hit model and signifies a role of the RES complex in brain associated phenotypes in humans [80]. We suggest *BUD13* regulates the expression of other neuronal genes through splicing and IR for the development of language abilities. Such regulatory effects could be involved in the delayed onset of language and the parallel growth trajectories of children with and without SLI, featuring a persistently lower level of language throughout childhood for the children with SLI [88]. Further investigation is needed concerning how IR machinery and splicing work in relation to Bud13.

Our study provides essential insight into the biological basis of SLI. However, it is vital to present some limitations of the current research, including our initial focus on a single family, the relatively small number of additional probands with SLI, the absence of behaviorally tested population-matched controls, and the utility of WES.

The investigation of a single family may limit the number of plausibly causal variants identified, and could introduce unique family variance leading to variant identification that may not be specific to the phenotype. However, the family-based filtering resulted in three variants with a reasonable cohesive variant level evidence of pathogenicity (Table 5). Crucially, the follow-up investigation provided gene level evidence in support of one gene, *BUD13*. These results support there is power in a single family-based investigation to target valuable further investigation in additional samples.

Our lack of behaviorally tested controls meant that we used SORVA to test the significance of the rate of variants in our identified genes, which utilizes the frequencies from the 1000 Genomes Project for comparison [48]. While there is not verified genetic ancestry information available for the additional probands with SLI, their self-reported race indicates a majority are White and likely have European ancestry (the KU cohort was collected from Kansas and Missouri). Therefore, we specified the parameters in SORVA to perform one comparison with the MAF in the global population and one with the MAF in the European subpopulation from the 1000 Genomes Project. The comparison with the global population yielded genome-wide significance, while the comparison with the European population did not. However, this does not mean *BUD13* is not significant for SLI in our sample, given that these comparisons were limited by the unverified genetic ancestry information of our additional probands and the unavailability of language phenotype data in the 1000 Genome Project samples.

Although WES provides an excellent source to study exonic variants, comparisons of variants identified through WES vs. whole genome sequencing (WGS) have shown differences in quality distribution [89]. These differences led to about 3% of coding variants being missed in the WES output, but not the WGS output, according to one analysis [89]. This could mean there are additional variants of interest in family 489 not observed in the WES output. We strongly suggest using WGS in the future, and selecting all the available samples from the family to increase the confidence of the filtered variants. Despite these limitations, our analysis indicates the utility of family-based studies for the identification of rare variants of a possibly large effect in SLI.

## 5. Conclusions

To better understand the genetic factors underlying SLI, we have done an initial survey using WES in SLI families, followed by candidate gene sequencing in unrelated individuals with this disorder. We identified three new candidate genes in which rare variants are co-segregated with SLI in family 489. Subsequently, the Sanger sequencing of these genes in other unrelated individuals with SLI identified additional genetic variants, some observed in multiple proband-ascertained families. We prioritized *BUD13* among other candidates based on the high frequency of individuals with SLI carrying genetic variants in this gene and its role in the mechanisms thought to be involved in neural phenotypes [79,80,90,91,92]. Genetic study of *BUD13* in SLI samples from other populations may provide more information about the genotype—phenotype relationship. Future studies could inform the extent to which *BUD13* or other suggested gene variants contribute to overall cases of SLI. It is plausible that variants in other genes (novel or previously suggested candidates) are present in individuals with SLI (not selected for WES) carrying *BUD13* variants. Our results suggest new gene targets for future studies in SLI, specifically *BUD13*, a component of the RES complex.

## Figures and Tables

**Table 1:** WES variants identified in candidate genes previously suggested for language and related phenotypes in family 489.

Gene	Genomic Position (hg19)	c.DNA Variant	AA Change	rsID	IDs of SLI Probands with Variant *n* = 175	MAF in gnomAD		In Silico Prediction Scores				
						Glob	Euro	SIFT	Poly Phen-2	Mutation assessor	PROVEAN	Mutation Taster
*KIAA0319*	Chr6:	c.2164G > A	p.Arg722Trp	rs113411083	NA	0.00275	0.0047	0.068	0.998	1.495	−3.28	101
	24566953							(T)	prob D	(low)	D	P
*FLNC*	Chr7:	c.6808G > A	p.Glu2270Lys	rs202223616	NA	0.00073	0.00168	1	0.371	1.23	−2.44	56
	128494547							(T)	B	(low)	N	DC
*NOP9*	Chr14:	c.62G > C	p.Arg21Pro	rs183868211	346, 353, 355, 411, 472	0.00936	0.02304	0.147	0.01	2.39	−0.94	103
	24769222							(T)	B	(med)	N	P

*KIAA0319*: NM_001168375, *FLNC*: NM_001458, *NOP9*: NM_174913; chr—chromosome; AA—amino acid; NA—not available; MAF—minor allele frequency presented from gnomAD v2.1.1 exomes; glob—global; Euro—European; in silico prediction scores: D—deleterious; T—tolerated; L—low (functional impact); M—medium (functional impact); N—neutral/(functional impact); H—high (functional impact); MutationTaster2—Grantham matrix score (0–215; amino acid comparison); P—polymorphism; DC—disease causing. MAF and in silico predictions scores acquired on or before 26 October 2021.

**Table 2:** Summary of rare variants co-segregating in family 489 according to a priori co-segregation criteria.

Gene	Genomic Position (hg19)	c.DNA Variant	AA Change	rsID	IDs of SLI Probands with Variant *n* = 175	MAF in gnomAD		In Silico Prediction Scores				
						Glob	Euro	SIFT	Poly Phen-2	Mutation Assessor	PROVEAN	Mutation Taster
*BUD13*	Chr11:	c.689G > A	p.Arg230Glu	rs139478949	NA	0.00002	0.00005	0.013	0.934	3.405	−2.51	43
	116633616							(D)	poss D	(med)	D	DC
*APLP2*	Chr11:	c.2041G > A	p.Val681Met	rs370970986	NA	0.00002	0.00002	0.39	0.94	1.1	−0.01	21
	130011820							(D)	poss D	(low)	N	P
*NDRG2*	Chr14:	c.143G > A	p.Gly48Asp	rs11552412	NA	NA	NA	0	1.00	3.445	−6.06	94
	21490631							(D)	prob D	(med)	D	DC

*BUD13*: NM_032725.4, *APLP2*: NM_001642, *NDRG2*: NM_201535; chr—chromosome; AA—amino acid, NA—not available; MAF—minor allele frequency presented from gnomAD v2.1.1 exomes; glob—global; Euro—European; in silico prediction scores: D—deleterious; T—tolerated; L—low (functional impact); M—medium (functional impact); N—neutral/(functional impact); H—high (functional impact); MutationTaster2: Grantham matrix score (0–215; amino acid comparison); P—polymorphism; DC—disease causing. MAF and in silico predictions scores acquired on or before 26 October 2021.

**Table 3:** Summary of rare variants in *APLP2* (NM_001642) identified through Sanger sequencing in probands with SLI.

Genomic Position (hg19) Chr11	c.DNA	AA Change	rsID	IDs of SLI Probands with Variant *n* = 175	MAF in gnomAD		In Silico Prediction Scores				
					Glob	Euro	SIFT	Poly Phen-2	Mutation Assessor	PROVEAN	Mutation Taster
129991652	c.660T > G	p.Asp220Glu	rs1063201	434	0.00006	0.00002	0.736	0	0.255	−0.59	45
							(T)	B	(neutral)	N	P
129992279	c.793G > A	p.Glu265Lys	NA	463	NA	NA	0.022	0	0.55	−1.54	56
							(D)	B	(neutral)	N	DC
130013358	c.*15G > A	3′UTR	rs201861910	447	0.001221	0.001972	NA	NA	NA	NA	NA

chr—chromosome; AA—amino acid, NA—not available; MAF—minor allele frequency presented from gnomAD v2.1.1 exomes; glob—global; Euro—European; in silico prediction scores: D—deleterious; T—tolerated; L—low (functional impact); M—medium (functional impact); N—neutral/(functional impact); H—high (functional impact); MutationTaster2: Grantham matrix score (0–215; amino acid comparison); P—polymorphism; DC—disease causing. MAF and in silico predictions scores acquired on or before 26 October 2021.

**Table 4:** Summary of rare variants in *BUD13* (NM_032725.4) identified through Sanger sequencing in probands with SLI.

Genomic Position (hg19) Chr11	c.DNA	AA Change	rsID	IDs of SLI Probands with Variant *n* = 175	MAF in gnomAD		In Silico Prediction Scores				
					Glob	Euro	SIFT	Poly Phen-2	Mutation Assessor	PROVEAN	Mutation Taster
116643617	c.64C > A	p.Ala22Ser	rs35585096	337, 455, 483, 405	0.023	0.000	0.112	0.578	2.44	−0.81	99
							(D)	poss D	(med)	N	P
116633875	c.430G > A	p.Arg144Cys	rs116087150	49324	0.000	0.000	0.045	1	2.81	−4.15	180
							(D)	prob D	(med)	D	DC
116633787	c.518T > C	p.Asp173Gly	rs1467808735	360	0.000	0.000	0.013	1	3.27	−4.47	94
							(D)	prob D	(med)	D	DC
116633724	c.581C > T	p.Arg194His	rs144776650	384, 422, 484	0.003	0.005	0.06	0.091	1.725	−3.73	29
							(T)	B	(low)	D	P
116633580	c.725C > A	p.Arg242Ile	rs11216131	500	0.001	0.001	0.002	0.999	3.58	−4.43	97
							(D)	prob D	(high)	D	DC
116633425	c.880C > G	p.Ala294Pro	rs1427011653	201	NA	NA	0.231	0.002	2.395	−0.75	27
							(T)	B	(med)	N	P
116633353	c.952A > T	p.Tyr318Asn	rs145410701	438	0.001	0.000	0.33	0.138	2.045	−1.26	142
							(T)	B	(med)	N	P
116631482	c.1223G > A	p.Pro408Leu	rs61730763	427	0.003	0.000	0.023	0.275	2.63	−7.04	98
							(D)	B	(med)	D	DC
116619178	c.*20G > A	3′UTR	rs145906707	431, 447	0.003	0.003	NA	NA	NA	NA	NA

chr—chromosome; AA—amino acid, NA—not available; MAF—minor allele frequency presented from gnomAD v2.1.1 exomes; glob—global; Euro—European; in silico prediction scores: D—deleterious; T—tolerated; L—low (functional impact); M—medium (functional impact); N—neutral/(functional impact); H—high (functional impact); MutationTaster2: Grantham matrix score (0–215; amino acid comparison); P—polymorphism; DC—disease causing. MAF and in silico predictions scores acquired on or before 26 October 2021.

## Data Availability

A portion of the dataset generated during and/or analyzed during the current study is available in the Supplementary Materials. Additional datasets generated during and/or analyzed during the current study are available from the corresponding author upon reasonable request.

## Supplementary Materials

The following are available online at https://www.mdpi.com/article/10.3390/brainsci12010047/s1. Figure S1: Additional BUD13 variants identified in probands with SLI and their family members. Note. Each pedigree is labelled on top with a unique ID. The cDNA and amino acid change for each variation is shown above the pedigree ID. Figure S2: Additional APLP2 variants identified in probands with SLI and their family members. Figure S3: Additional NOP9 variants identified in probands with SLI and their family members. Note. Each pedigree is labelled on top with a unique ID. The cDNA and amino acid change for each variation is shown above the pedigree ID. The plus symbol indicates the individual carries the variant allele. Supplementary Spreadsheets: Tables S1–S7: Lists of prioritized rare WES variants in a sequential filtering procedure in family 489 and those variants filtered in the linkage regions suggested in family 489 and 113 previously suggested candidate genes. Table S1. 137 variants: Rare exonic and splicing variants under autosomal dominant inheritance, absent in unaffected sibling (ID: 4894). Table S2. 84var CADDfilter: List of 84 variants with an unknown CADD raw score or score > 1.0. Table S3. 76var: GERP filter: List of 76 variants when with negative GERP++ RS score removed. Table S4. 54var SegDupl filter: List of 55 variants when variants in segmentally duplicated regions were removed. Table S5. 89var 14q locus: All 89 variants within the suggestive linkage locus at chromosome 14q11.2–13.3. Table S6. 14var 13q locus: All 14 variants within the suggestive linkage locus at chromosome 13q14.12–14.3. Table S7. 12var cand-genes: All 12 variants within the 113 cross-referenced candidate genes previously suggested for language and related phenotypes. Table S8. Candidate Genes: All 113 candidate genes previously suggested for language and related phenotypes used for cross-referencing under the candidate gene filtering prioritization workflow.
