# Supplementary figures and images for: Family-Based Whole-Exome Analysis of Specific Language Impairment (SLI) Identifies Rare Variants in BUD13, a Component of the Retention and Splicing (RES) Complex
Source: Brain Sci. 2021 Dec 30;12(1):47. doi: 10.3390/brainsci12010047 (PMC8773923; doi:10.3390/brainsci12010047)

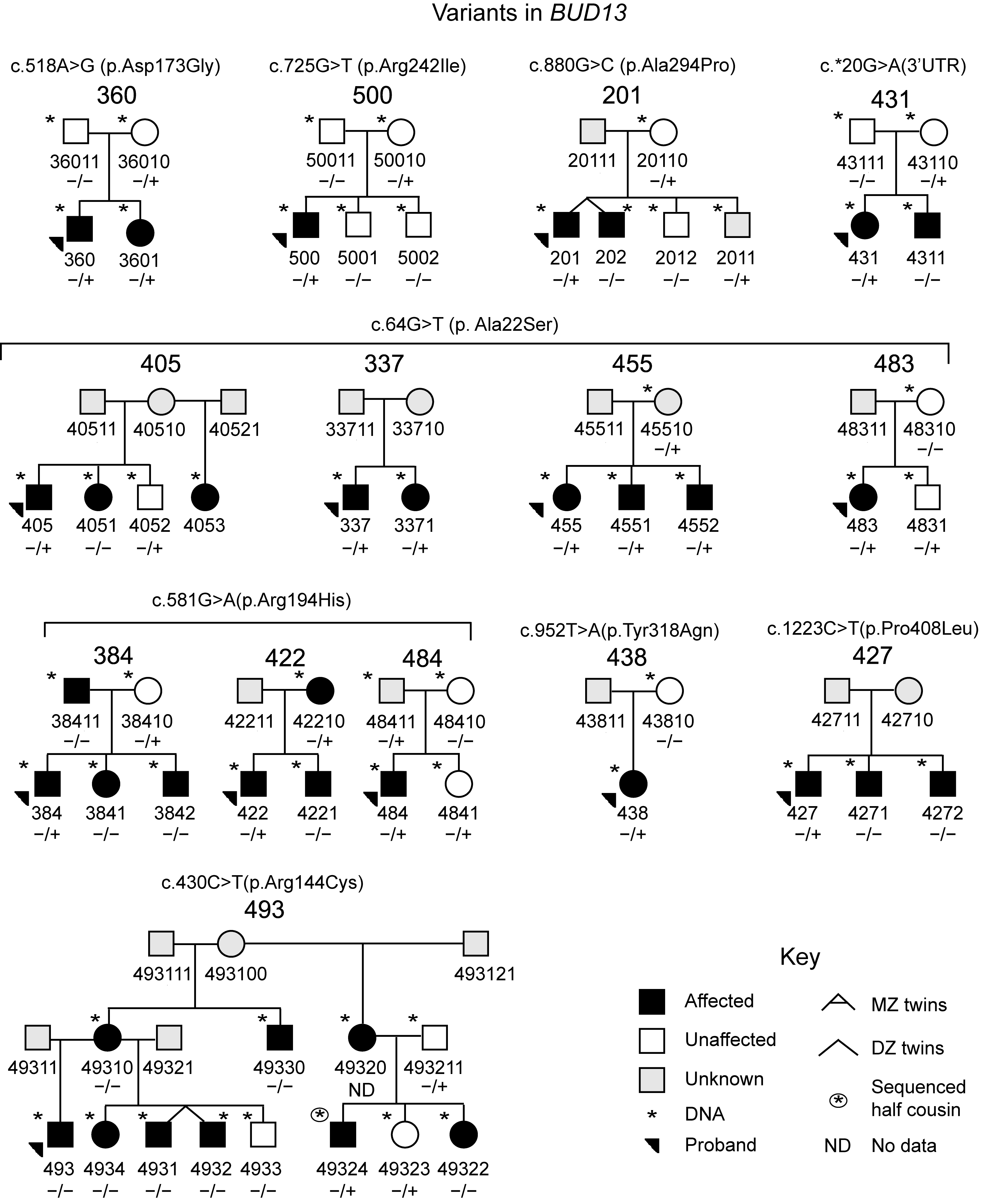

Supplement: Supplementary file 1 [file brainsci-12-00047-s001.zip › Andres_et-al_2021 Figure_S1.tif]

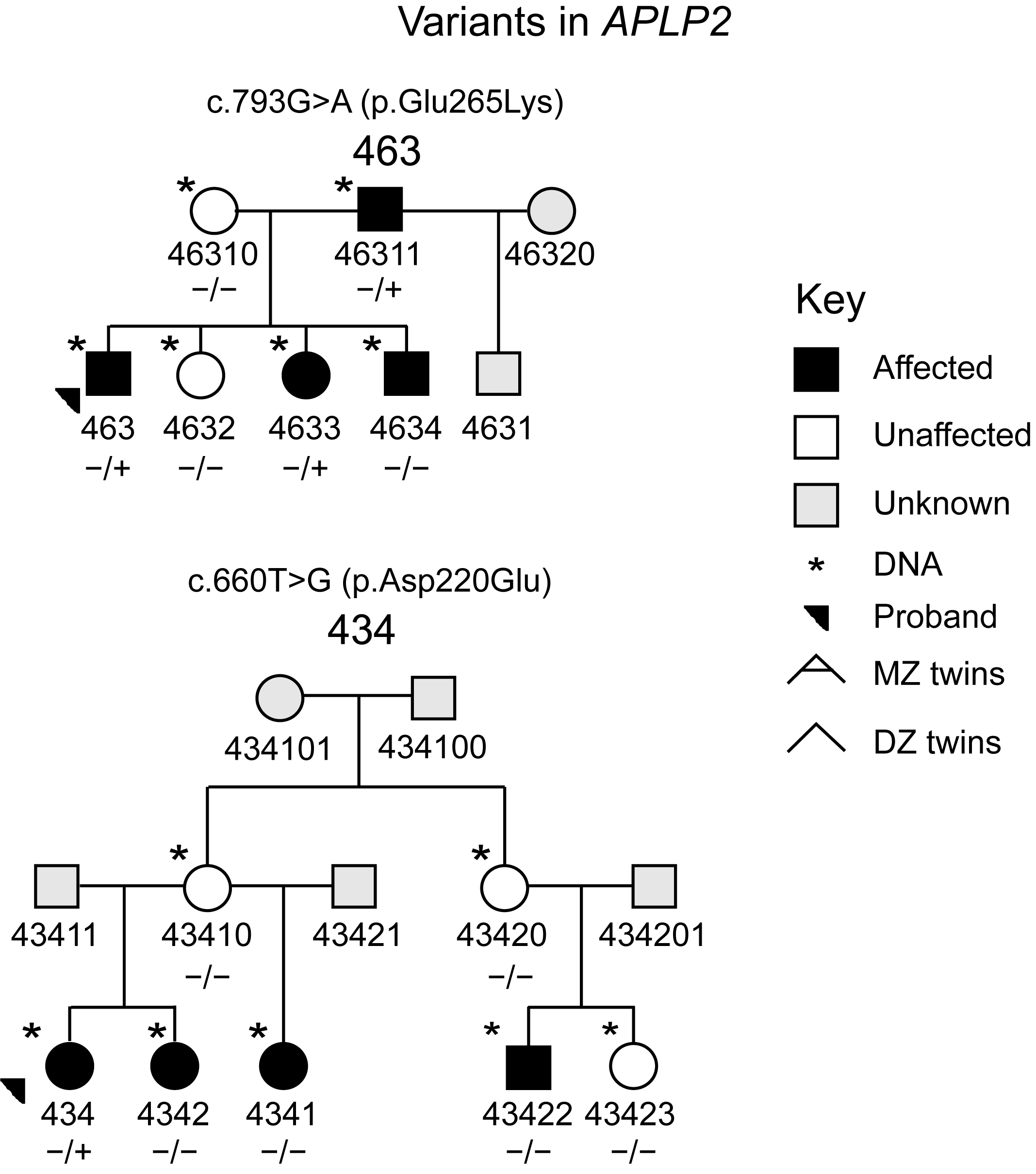

Supplement: Supplementary file 1 [file brainsci-12-00047-s001.zip › Andres_et-al_2021 Figure_S2.tif]

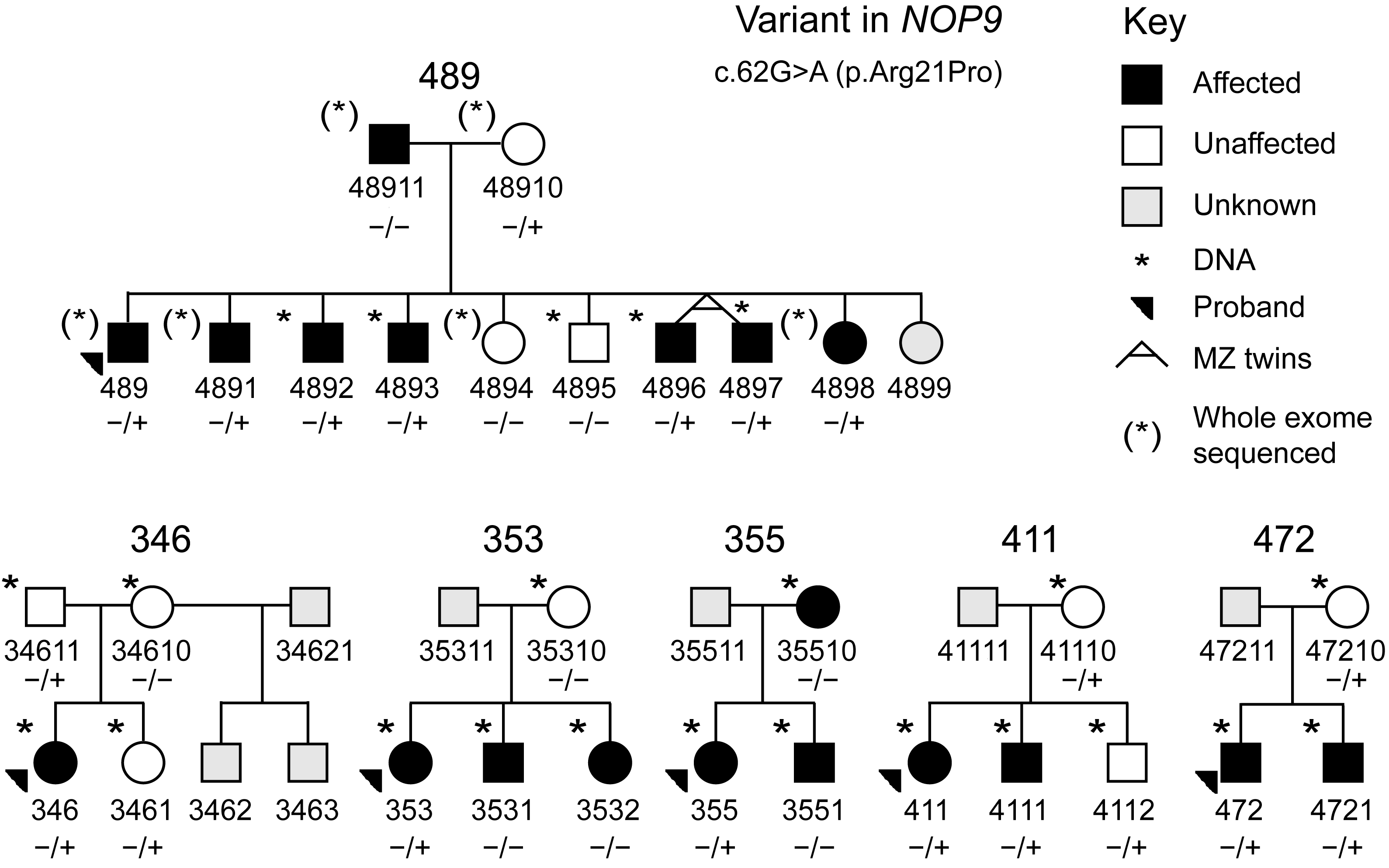

Supplement: Supplementary file 1 [file brainsci-12-00047-s001.zip › Andres_et-al_2021 Figure_S3.tif]
